# Reduced *SKP1* Expression Induces Chromosome Instability through Aberrant Cyclin E1 Protein Turnover

**DOI:** 10.3390/cancers12030531

**Published:** 2020-02-25

**Authors:** Laura L. Thompson, Allison K. Baergen, Zelda Lichtensztejn, Kirk J. McManus

**Affiliations:** 1 Department of Biochemistry & Medical Genetics, University of Manitoba, Winnipeg, MB R3E 0J9, Canada; Thompson.Laura1@mayo.edu (L.L.T.); baergeak@myumanitoba.ca (A.K.B.); Zelda.Lichtensztejn@umanitoba.ca (Z.L.); 2 Research Institute in Oncology & Hematology, CancerCare Manitoba, Winnipeg, MB R3E 0V9, Canada

**Keywords:** cancer, genome instability, chromosome instability, SCF complex, SKP1, cyclin E1, chromothripsis, single-cell quantitative imaging microscopy

## Abstract

Chromosome instability (CIN), or progressive changes in chromosome numbers, is an enabling feature of many cancers; however, the mechanisms giving rise to CIN remain poorly understood. To expand our mechanistic understanding of the molecular determinants of CIN in humans, we employed a cross-species approach to identify 164 human candidates to screen. Using quantitative imaging microscopy (QuantIM), we show that silencing 148 genes resulted in significant changes in CIN-associated phenotypes in two distinct cellular contexts. Ten genes were prioritized for validation based on cancer patient datasets revealing frequent gene copy number losses and associations with worse patient outcomes. QuantIM determined silencing of each gene-induced CIN, identifying novel roles for each as chromosome stability genes. SKP1 was selected for in-depth analyses as it forms part of SCF (SKP1, CUL1, FBox) complex, an E3 ubiquitin ligase that targets proteins for proteolytic degradation. Remarkably, *SKP1* silencing induced increases in replication stress, DNA double strand breaks and chromothriptic events that were ascribed to aberrant increases in Cyclin E1 levels arising from reduced *SKP1* expression. Collectively, these data reveal a high degree of evolutionary conservation between human and budding yeast CIN genes and further identify aberrant mechanisms associated with increases in chromothriptic events.

## 1. Introduction

Chromosome instability (CIN) is a form of genome instability characterized by an increase in the rate at which whole chromosomes (numerical CIN) or large chromosomal fragments (structural CIN) are gained or lost [1]. Due to the continual changes in chromosome (genetic) complements, CIN is synonymous with cell-to-cell heterogeneity, which can confer growth and survival advantages that promote oncogenesis. Decades of fundamental and clinical studies have established strong associations between CIN and cancer. For example, CIN drives cellular transformation, promotes intratumoral heterogeneity, enhances metastatic potential, induces multi-drug resistance and is associated with poor patient outcomes (reviewed in [2,3,4]). Despite these associations, the molecular determinants (i.e., the aberrant genes and pathways) causing CIN remain poorly understood in humans. Accordingly, identifying novel CIN genes and characterizing their mechanism of actions will not only provide fundamental insight into disease pathogenesis, but may reveal genes with diagnostic, prognostic and/or therapeutic potential in cancer.

In 2011, Stirling et al. [5] determined that ~11.5% of all budding yeast genes are CIN genes. More specifically, they showed that deletion or reduced expression of 692/6000 genes resulted in gains or losses of whole chromosomes or large chromosomal fragments. The diversity of CIN genes identified encode a myriad of distinct biological pathways, including those with intuitive links to CIN, such as DNA replication, DNA repair and mitotic spindle assembly, and those with less intuitive links, including glycan anchor biosynthesis, vesicular trafficking and proteasomal degradation. If the high frequency of CIN genes is maintained in humans, up to 2300 CIN genes (11.5% of 20,000 total genes) may exist within the human genome; however, only a small proportion of human CIN genes have been identified and characterized to date. As many yeast CIN genes have human orthologs that encode functions within evolutionarily conserved biological pathways, we believe cross-species candidate gene approaches can assist in the identification of novel human CIN genes with pathogenic implications for cancer development and progression.

In this study, we sought to gain novel insight into the mechanisms causing CIN in cancer. By coupling cross-species approaches and single-cell quantitative imaging microscopy (scQuantIM), we determined that ~90% (148 of 164) of genes assessed induced CIN phenotypes in two distinct cellular contexts. Subsequent direct tests involving 10 prioritized genes (*ARL2*, *BUB3*, *DSN1*, *NUF2*, *SPC24*, *GART*, *SHMT2*, *GARS*, *PIGS* and *SKP1*) encoding a diverse array of biological functions and exhibiting frequent gene copy number losses and links to worse patient outcomes were performed that validated each as a novel CIN gene. Subsequent in-depth analyses revealed that *SKP1* silencing, a component of the SCF (SKP1, CUL1, FBox) E3 ubiquitin ligase complex, corresponded with increases in CIN phenotypes, replication stress, DNA double-strand breaks (DSBs), numerical changes in chromosome numbers and chromothriptic events that were rescued following co-silencing with Cyclin E1 (CCNE1), a substrate of the SCF complex. These results highlight a high degree of evolutionary conservation in CIN genes and are consistent with reduced *SKP1* expression or diminished SCF activity being pathogenic events driving cancer development and progression.

## 2. Results

### 2.1. Cross-Species Analyses and Single-cell Quantitative Imaging Microscopy Identify Putative Human CIN Genes

In 2011, Stirling and colleagues [5] identified 692 *Saccharomyces cerevisiae* genes that when deleted or aberrantly expressed induced CIN by at least one of three yeast CIN assays. As many biological processes are conserved across evolution, we reasoned that CIN genes are also likely to be conserved. Accordingly, a cross-species candidate gene approach was employed to identify the most promising candidates to screen in a human context. Of the 692 yeast CIN genes identified [5], 485 were determined to have sequence and/or functional human orthologues. From these, 164 human genes (Table S1) were subsequently prioritized based on strong corresponding yeast meta-scores (i.e., strong CIN phenotypes by one or more CIN assays), and that they were not well-established human CIN genes based on available literature.

**Figure 1:**
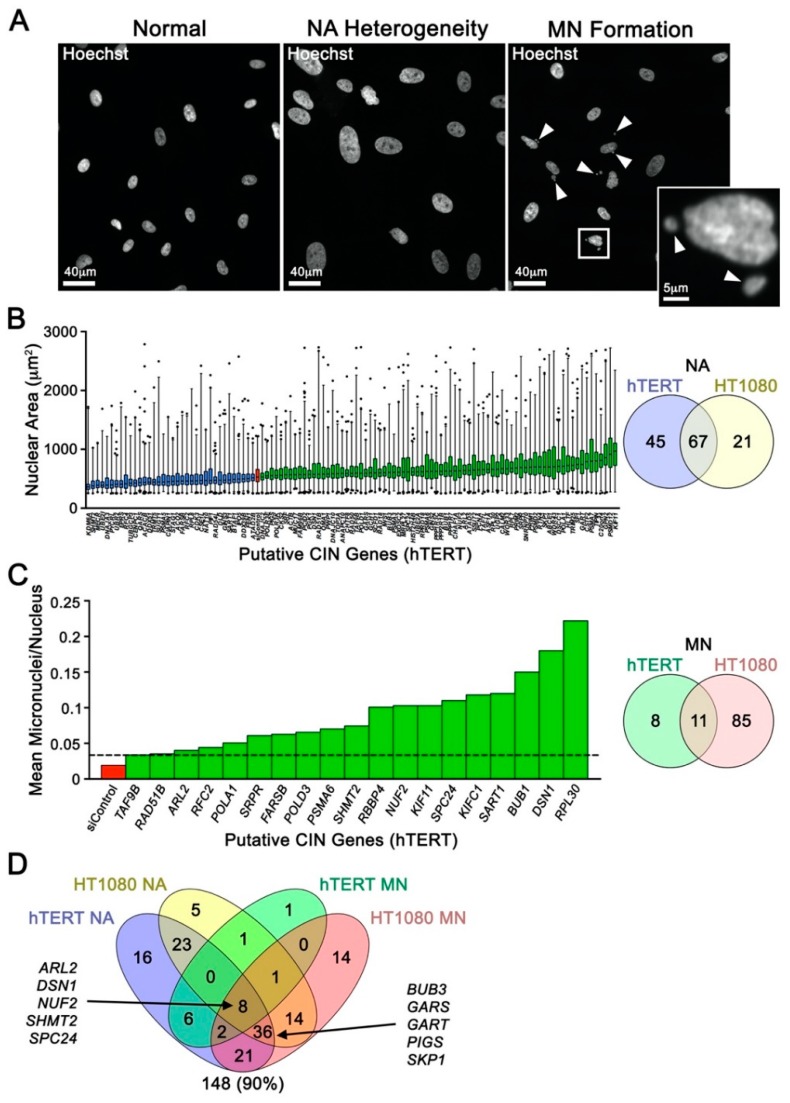
Single-Cell Quantitative Imaging Microscopy Uncovers Putative Chromosome Instability (CIN) Genes. (**A**) Representative fluorescent microscopy images highlighting nuclear area (NA) size heterogeneity (center) and micronucleus formation (right; arrowheads) that accompany silencing of a CIN gene relative to karyotypically stable cells (left). (**B**) Box-and-whisker graph (left) displaying 1st–99th percentiles (whiskers) and the interquartile range (25th, 50th, and 75th percentiles) of NA data for putative CIN gene identified from the single-cell quantitative imaging microscopy (scQuantIM) screen performed in hTERT. Note that only genes inducing statistically significant decreases or increases in cumulative NA distributions relative to control (siControl; red) are presented and are indicated in blue and green, respectively. Venn diagram (right) displaying the numbers of putative CIN genes identified in the scQuantIM NA screen in both hTERT and HT1080. (**C**) Bar graph (left) presenting the normalized number of micronuclei/nucleus following silencing of candidate CIN genes (green) in hTERT. Only those genes inducing micronucleus (MN) formation above the minimum threshold (mean + 2 SD of siControl; dashed line) are presented. Venn diagram (right) displaying the numbers of putative CIN genes identified in the scQuantIM MN formation screen in both hTERT and HT1080. (**D**) Venn diagram reveals 148 putative CIN genes were identified from the NA and MN formation screens performed in hTERT and HT1080. The 10 genes selected for subsequent validation are indicated.

To gain novel insight into the genes and pathways regulating chromosome stability in humans, these 164 candidate genes, along with positive (*SMC1A*) and negative controls (siControl) [6,7], were subjected to a single-cell quantitative imaging microscopy (scQuantIM) screen in two distinct and karyotypically stable human cell lines (hTERT and HT1080). These genes, along with positive (*SMC1A*) and negative (siControl) controls, were silenced using a custom-arrayed siGENOME (Dharmacon) library. Putative CIN genes were identified as those inducing significant changes in surrogate markers of CIN, namely changes in nuclear areas (NAs) and micronucleus (MN) formation (Figure 1A). Conceptually, changes in NAs correspond with large scale changes in DNA/chromosome complements, while micronuclei are hallmarks of CIN that typically arise due to chromosome mis-segregation events and defects in DSB repair (reviewed in [8]). In hTERT cells, scQuantIM identified 112 genes (68% of genes screened) that induced significant changes in cumulative NA frequency distributions (Kolmogorov-Smirnov (KS) test; *p*-value < 0.01) following silencing relative to siControl (Figure 1B; Table S2), while 19 genes (12%) induced significant increases in MN formation (Figure 1C; Table S3). Similarly, 88 (54%) and 96 (59%) genes silenced in HT1080 cells induced significant changes in NAs and MN formation, respectively (Figure 1B,C; Tables S4 and S5). Although the underlying mechanism(s) accounting for the differences in gene complements observed within the two cell lines was not determined, it likely stems from their distinct genetic contexts—HT1080 are a fibrosarcoma line harboring genetic alterations in the DNA damage response (WRN and ERCC5) and apoptotic (BID) pathways [9], while hTERT are a “normal” immortalized fibroblast. In total, 148 (90%) genes were identified as putative CIN genes (Figure 1D) and although this is a high recapitulation rate, it is not unexpected as the human genes were identified from established CIN genes in yeast [5]. These findings underscore the high degree of evolutionary conservation that exists between human and yeast CIN genes.

### 2.2. Putative CIN Genes Exhibit Frequent Copy Number Losses that may Be Pathogenic in Cancer

**Figure 2:**
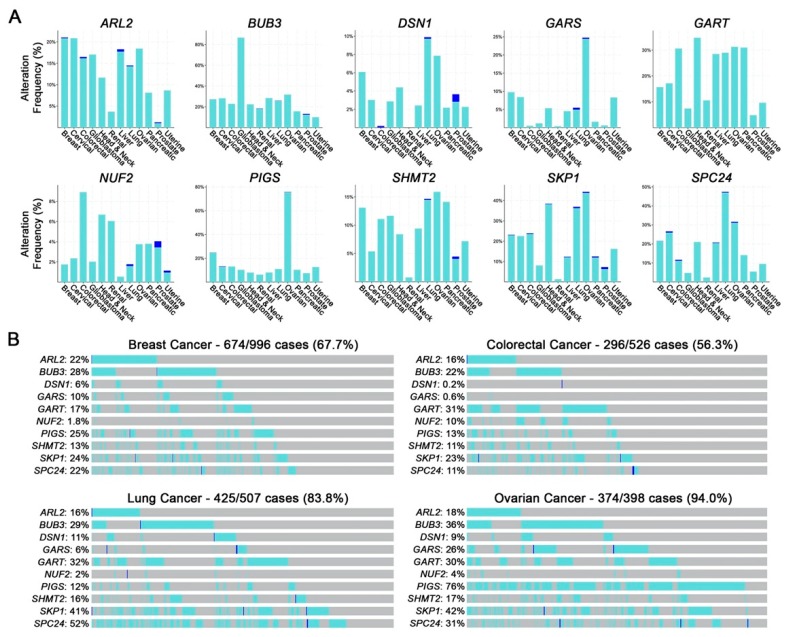
Hypomorphic CIN Gene Alterations are Frequently Observed in Cancer. (**A**) Frequency of gene copy number alterations in 12 common cancer types with shallow (heterozygous) and deep (homozygous) deletions indicated in aqua and blue, respectively. Data were extracted from publicly available TCGA datasets [19]. (**B**) Cumulative frequency of shallow and deep deletions for all 10 genes in breast (67.7%), colorectal (56.3%), lung (83.8%) and ovarian (94.0%) cancers. The frequency of individual gene alterations for each cancer is indicated.

Of the 148 putative CIN genes identified above, 10 (Figure 1D) were prioritized for subsequent validation based on the conservation of CIN phenotypes (i.e., identified by a minimum 3 of 4 assays), the magnitude of the CIN phenotypes induced and their potential relevance in cancer. Genes were also selected to represent a diverse array of gene ontology groups. *ARL2*, *BUB3*, *DSN1*, *NUF2* and *SPC24* encode functions in mitosis with specific roles in mitotic spindle assembly, microtubule dynamics, and kinetochore-microtubule attachments [10,11,12], while *GART* (de novo purine biosynthesis [13]), SHMT2 (de novo thymidylate biosynthesis [14]), GARS (glycyl-tRNA synthesis [15]), *PIGS* (GPI-anchor biosynthesis [16]) and *SKP1* (ubiquitin-mediated proteasomal degradation [17]) encode functions that are not immediately linked to CIN and were purposefully selected to gain insight into novel mechanisms responsible for CIN. Using publicly available patient-derived datasets from The Cancer Genome Atlas (TCGA) Network [18,19], we determined that all 10 genes are frequently altered in cancer, with heterozygous (shallow) and homozygous (deep) deletions occurring in at least 12 common cancer types (Figure 2A). Further, the cumulative frequency of heterozygous and homozygous loss in four common cancer types is >50% of all cases, ranging from ~56% in colorectal cancer to ~94% in ovarian cancer (Figure 2B), implicating reduced expression as a potential pathogenic event in cancer. This possibility is further strengthened by the observation that reduced mRNA expression of each gene corresponds with significantly worse overall patient survival in various cancer types (Figure S1) [19,20]. Collectively, these findings coupled with those of the initial CIN screen suggest reduced expression may induce CIN and drive cancer development, which warrants further study.

### 2.3. Reduced Gene Expression Drives Increases in Chromosome Aberrations

To firmly establish the CIN phenotypes observed above were due to on-target effects, a distinct set of ON-TARGETplus (Dharmacon, Lafayette, CO, USA) siRNA duplexes were used for validation. To further show that the results are cell type independent, work was expanded into a third karyotypically stable cell line, HCT116, which has been used extensively in similar CIN-based studies [6,21,22,23]. Western blots established the silencing efficiencies of all 10 genes (Figure S2) and scQuantIM validated the results of the initial hTERT screen. Further, gene silencing in HCT116 corresponded with statistically significant increases in nuclear areas, and 2.5- to 14.6-fold increases in MN formation for all genes, with the exception of *GARS* (0.9-fold), *GART* (1.5-fold) and *SHMT2* (0.2-fold) (Table S6). Collectively, these results show that reduced expression of all 10 genes induces increases in CIN phenotypes that are independent of cell type.

**Figure 3:**
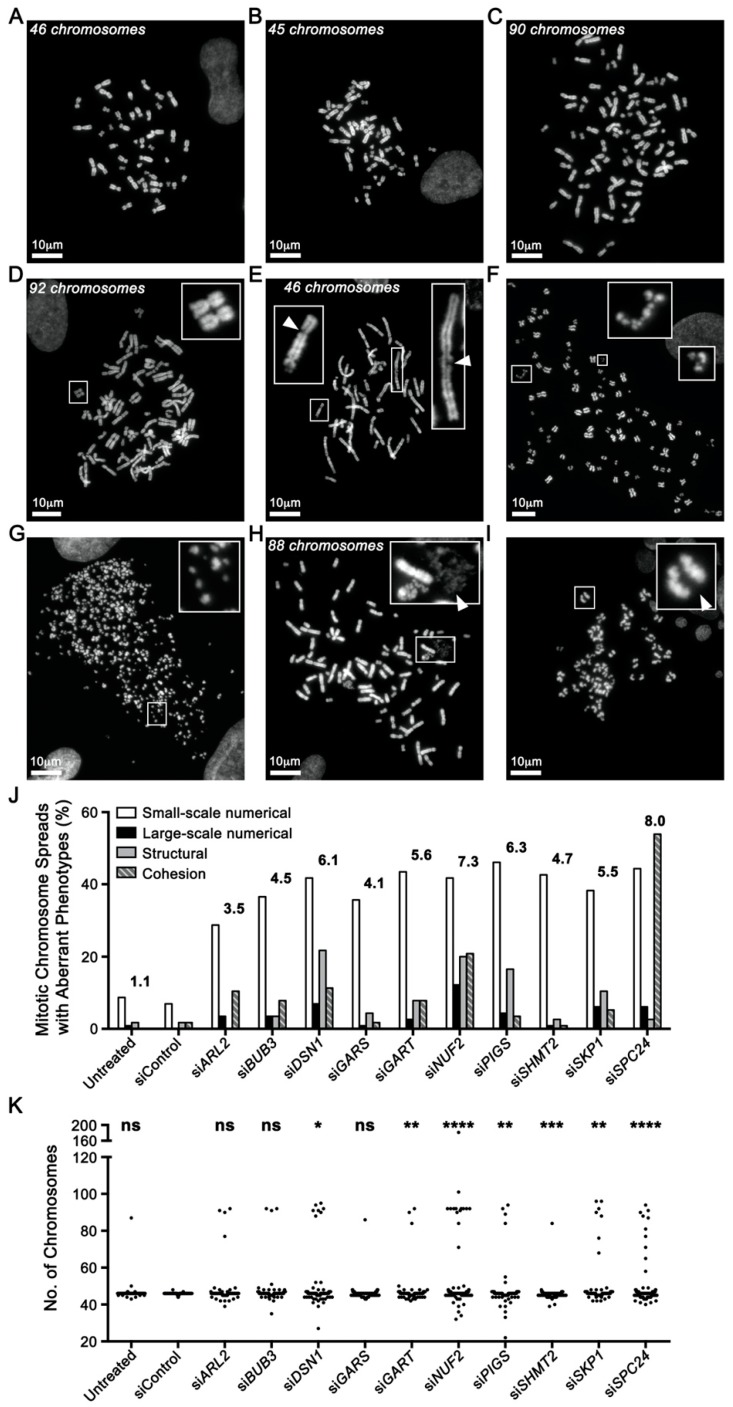
Gene Silencing Induces Numerical, Structural and Cohesion Chromosome Defects. (**A**) Representative mitotic chromosome spread from hTERT cells exhibiting the expected number (46) of chromosomes. (**B**) Aberrant chromosome spread displaying a small-scale numerical defect involving <15 chromosomes. (**C**) Large-scale numerical defect involving ≥15 chromosomes. (**D**) Endoreduplication resulting in paired homologous chromosomes (magnified inset) and a large-scale numerical defect. (**E**–**G**) Chromosome breakages (i.e., structural defects) increasing in severity from DNA double-strand breaks (DSBs) on individual chromatids (**E**; arrowheads), to extensive breaks along the length of the chromosomes (**F** and **G**; magnified insets). (**H**) Chromosome decompaction classified as a structural defect. Arrowhead highlights a region of chromosome decompaction. (**I**) Sister chromatid cohesion defects, where a spatial separation between sister chromatids is visually apparent at the primary constriction (centromere). (**J**) Bar graph displaying increases in the various chromosome defects indicated following gene silencing. The numbers above the bars identify the fold increase in total aberrant events for each gene relative to control (siControl). (**K**) Dot plot presenting the number of chromosomes enumerated following gene silencing. Kolmogorov–Smirnov (KS) tests reveal significant changes in the cumulative distribution frequencies as indicated (ns = not significant, * = *p*-value < 0.05, ** = *p*-value < 0.01, *** = *p*-value < 0.001, and **** = *p*-value < 0.0001).

To gain insight into the underlying mechanism(s) causing the CIN phenotypes, mitotic chromosome spreads were generated and assessed for numerical and structural defects (Figure 3). For simplicity, numerical chromosome defects were classified as either small (<15 chromosomes; Figure 3B) or large (≥15 chromosomes; Figure 3C,D) scale changes, while structural defects included chromosome breakages (DNA DSBs (Figure 3E,F) or chromosome shattering (Figure 3G)), chromosome compaction/condensation defects (Figure 3H) and sister chromatid cohesion defects [6] (Figure 3I). Overall, gene silencing corresponded with a 3.5- to 8.0-fold increase in the collective frequency of numerical and structural defects within hTERT cells (Figure 3J), with *DSN1*, *GART*, *NUF2*, *PIGS*, *SHMT2*, *SKP1*, and *SPC24* silencing corresponding with significant differences in the cumulative distribution of chromosome numbers (Figure 3K; Table S6). Similarly, gene silencing in HCT116 cells corresponded with a 3.9- to 5.9-fold increase in numerical and structural defects (Figure S3A; Table S6) with significant changes in the cumulative chromosome number distributions occurring for each gene (Figure S3B). As above, the more pronounced phenotypes typically occurring in HCT116 likely reflects their distinct genetic status, particularly since they harbor a *MLH1* deficiency rendering them DNA mis-match repair defective [9]. Remarkably, although chromosome breakages (Figure 3E) occurred for each gene, silencing of *DSN1*, *NUF2* and *SKP1* induced extensive chromosomal breakages or “shattering” (Figure 3F,G) that are indicative of replication stress and chromothripsis, a catastrophic chromosome fragmentation event resulting in complex chromosome rearrangements that induce tumorigenesis [24,25]. Collectively, these show that reduced gene expression corresponds with increases in numerical and structural defects, further confirming their status as novel CIN genes.

### 2.4. Reduced SKP1 Expression Induces Replication Stress and DNA Double-strand Breaks

**Figure 4:**
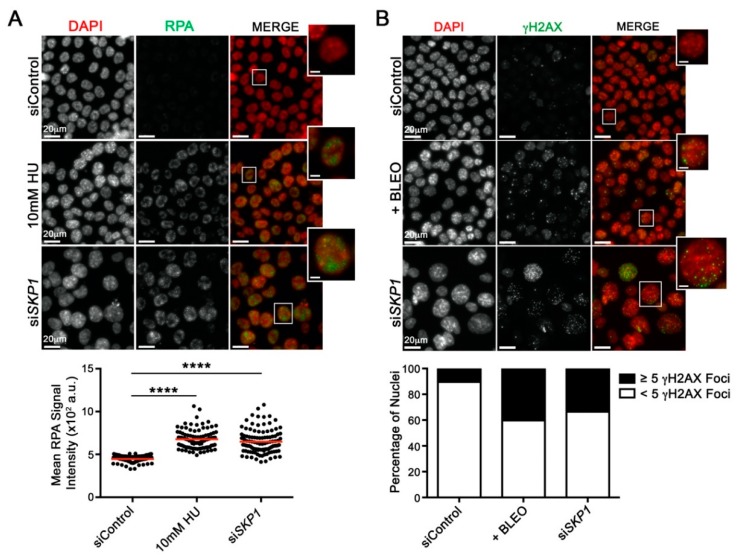
*SKP1* Silencing Induces Replication Stress and DNA Damage. (**A**) Representative maximum intensity projections (top; micrographs) showing visual increases in Replication Protein A (RPA) labeling in HCT116 cells following *SKP1* silencing relative to siControl; hydroxyurea (HU) is a positive control. Note that all images were acquired using identical exposure times. For illustrative purposes, a single nucleus is magnified from each condition (bounding box; scale bar represents 5 µm). ScQuantIM (bottom; dot plot) reveals significant increases in mean RPA signal intensity following *SKP1* silencing (or HU treatment) relative to siControl (Student’s *t*-tests; **** = *p*-value < 0.0001). (**B**) Qualitative images (top; micrographs) showing visual increases in γH2AX labeling in HCT116 cells following *SKP1* silencing relative to siControl; bleomycin (BLEO) is a positive control. Note that all images were acquired using identical exposure times. A single nucleus is magnified from each condition (bounding box; scale bar represents 5 µm). ScQuantIM (bottom; bar graph) reveals a 3.2-fold increases in the percentage of nuclei harboring ≥5 γH2AX foci (black) following *SKP1* (33.5%) silencing relative to siControl (10.4%); bleomycin treatments increased 3.9-fold (40.3%).

As the molecular origins of CIN and chromothripsis remain largely unknown (reviewed in [26,27,28]), SKP1 was purposefully selected for further in-depth study due to the conserved aberrant phenotypes observed in all three cell lines. SKP1 is the adaptor component of the SCF E3 ubiquitin ligase complex, which regulates polyubiquitination and proteasomal degradation of a myriad of downstream substrates [29]. Although the relationship between SKP1, the SCF complex and CIN is not be immediately clear, loss of SKP1 and SCF function presumably adversely impacts substrate turnover, which includes proteins involved in DNA replication and DSB repair [30,31]. To determine whether reduced *SKP1* expression induces replication stress, cells were immunofluorescently labeled for Replication Protein A (RPA), a single-stranded DNA binding protein that increases in abundance upon replication stress [32]. As shown in Figure 4A, *SKP1* silencing corresponded with a significant increase (Student’s *t*-test, *p*-value < 0.0001) in mean RPA signal intensity, similar to that observed following hydroxyurea (HU) treatment, a positive control [33]. Since replication stress can induce DSBs following replication fork collapse, we assessed whether reduced *SKP1* expression corresponded with increases in γH2AX foci, a surrogate marker of DSBs [34]. Figure 4B shows there was a 3.4-fold increase in the frequency of cells harbouring ≥5 γH2AX foci (34%) following *SKP1* silencing relative to siControl (10%), which is similar to that observed within the bleomycin treated (positive control) cells (40%). Collectively, these data show that *SKP1* silencing induces replication stress and DSBs that are consistent with both CIN and chromothripsis [24,35,36]. These findings also implicate the dysregulation of DNA replication and repair to aberrant SCF complex function and the impaired turnover of downstream substrates involved in those processes.

### 2.5. SKP1 Silencing Underlies Aberrant Increases in the Oncoprotein Cyclin E1

Cyclin E1 (CCNE1) is a prototypic cell cycle protein that regulates the G1 to S-phase transition, DNA replication, DNA repair and genome stability (reviewed in [37]). Cyclin E1 protein levels are regulated by the SCF complex [38,39,40], and although many studies have established strong causal links between genomic amplification of *CCNE1* and many cancer types (e.g., breast, liver and ovarian) [41,42,43,44], there is a paucity of information pertaining to the misregulation and ensuing overabundance of CCNE1 protein levels resulting from defects in the SCF complex. Gene amplification and ectopic *CCNE1* overexpression induces CIN [39] and is associated with aggressive cancers, chemotherapeutic resistance and poor prognosis [45]. Although many SCF complex substrates remain to be identified, CCNE1 is an established SCF substrate; however, the impact reduced *SKP1* expression and SCF targeting have on CCNE1 protein levels and CIN has never been assessed.

**Figure 5:**
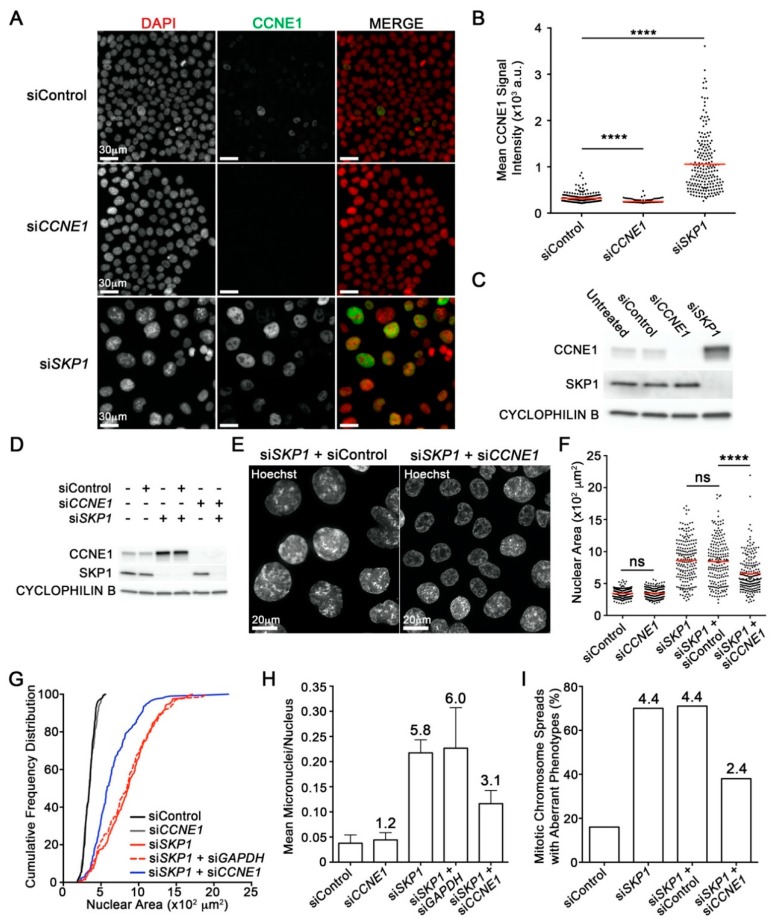
*SKP1* Silencing Induces Increases in Cyclin E1 (CCNE1) Levels that Contribute to CIN. (**A**) Qualitative micrographs showing visual increases in CCNE1 (green) levels following *SKP1* silencing in HCT116 cells. Images were acquired using identical exposure times. (**B**) Dot plot showing statistically significant increases in mean CCNE1 signal intensity (red line) in si*SKP1* cells relative to siControl (Student’s *t*-test, **** = *p*-value < 0.0001). (**C**) Western blot confirms CCNE1 protein levels increase following *SKP1* silencing. See Figure S4A for detailed information. (**D**) Western blot showing effective individual and co-silencing of SKP1 and CCNE1 in HCT116 cells. See Figure S4B for detailed information. (**E***)* Qualitative images showing visual decreases in NAs (phenotypic rescue) in co-silenced (si*SKP1* + si*CCNE1*) cells relative to control (si*SKP1* + siControl) cells. (**F**) ScQuantIM reveals significant decreases in mean NAs (red line) following co-silencing (si*SKP1* + si*CCNE1*) relative to control (si*SKP1* + siControl) (Student’s *t*-test, *p*-value <0.0001). (**G**) Co-silencing (si*SKP1* + si*CCNE1*) induces a significant phenotypic rescue (leftward shift) towards a smaller NA distribution relative to si*SKP1* or si*SKP1* + siControl (KS test, *p*-value <0.0001). (**H**) Bar graph showing a decrease in mean MN formation (+ SD) following co-silencing (si*SKP1* + si*CCNE1*) relative to controls (si*SKP1* or si*SKP1* + siControl); fold increase relative to siControl is presented above each bar. (**I**) Bar graph showing a decrease in the percentage of aberrant mitotic chromosome spreads following co-silencing relative to si*SKP1* or si*SKP1* + siControl; fold increase is indicated.

Before determining whether the CIN phenotypes observed following *SKP1* silencing were the result of reduced Cyclin E1 turnover, we first established Cyclin E1 as a bone fide substrate of the SCF complex. Accordingly, scQuantIM and Western blot analyses were employed that confirmed statistically significant increases in mean Cyclin E1 protein levels within *SKP1* silenced cells relative to controls (Figure 5A–C). Next, to determine whether the lack of CCNE1 protein turnover accounts for the CIN phenotypes observed following *SKP1* silencing, genetic rescue experiments were performed. More specifically, we sought to determine whether co-silencing *SKP1* and *CCNE1* (si*SKP1* + si*CCNE1*) would limit the CIN phenotypes observed when *SKP1* was silenced alone (si*SKP1*) or in combination with a negative control (si*SKP1* + siControl). Overall, co-silencing (si*SKP1* + si*CCNE1*) corresponded with visually striking and statistically significant decreases in mean NAs relative to si*SKP1* or si*SKP1* + siControl (Figure 5D,F). Furthermore, co-silencing also reduced the NA distributions away from the si*SKP1* or si*SKP1* + siControl conditions towards those of the negative controls (siControl or si*CCNE1*) (Figure 5G). Finally, scQuantIM approaches also revealed a 52% decrease in mean micronucleus formation and a similar decrease in the number of aberrant mitotic chromosome spreads following co-silencing (Figure 5H,I). Collectively, these data show that reduced *SKP1* expression leads to an increase in CCNE1 protein levels that are consistent with a lack of CCNE1 protein turnover, which accounts for a large portion of the aberrant increases in NAs, MN formation and chromosome aberrations observed. Accordingly, these data identify aberrant CCNE1 protein turnover resulting from reduced *SKP1* expression as a novel mechanism driving CIN that is independent of genomic amplification of *CCNE1*.

## 3. Discussion

Due to the strong relationship between CIN and cancer, we sought to gain insight into the molecular determinants of CIN in humans using a cross-species approach. Here, we identified and screened 164 human candidate genes, and determined 148 induced significant changes in NAs and/or MN formation. This 90% recapitulation rate reveals a high degree of evolutionary conservation between human and yeast CIN genes. Next, 10 genes were prioritized for subsequent study as in silico analyses determined that each exhibits frequent losses (heterozygous and homozygous) in 12 common cancer types, while low mRNA expression levels correlated with poor patient survival [19,20]. In addition to increases in NAs and MN formation, gene silencing also induced increases in numerical and/or structural chromosome defects. Of particular note, *DSN1*, *NUF2* and *SKP1* silencing resulted in DSBs and chromosome shattering phenotypes indicative of replication stress and suggestive of chromothripsis. *SKP1* silencing was subsequently shown to cause increases in replication stress and DSBs that are consistent with replication fork collapse. Finally, because SKP1 and the SCF complex regulate CCNE1 protein turnover, phenotypic rescue experiments were performed that established a causal link between reduced *SKP1* expression, increased CCNE1 protein levels and CIN. Collectively, this work has expanded our fundamental understanding of the molecular determinants causing CIN, some of which may have pathogenic and clinical implications in cancer.

Collectively, *ARL2*, *BUB3*, *DSN1*, *NUF2*, and *SPC24* encode functions in kinetochore/mitotic spindle assembly and chromosome dynamics [10,11,12]. As defective kinetochore structure/capture and aberrant chromosome dynamics underlie chromosome mis-segregation events, we expected our screen to uncover genes encoding proteins within these pathways; however, as reduced expression also induced increases in cohesion defects, these genes also appear to encode functions impacting sister chromatid cohesion under normal conditions. On the other hand, *GART*, *SHMT2*, *GARS*, *PIGS* and *SKP1* encode functions in pathways with less intuitive links to CIN. Conceptually, reduced expression of *GART* [13] or *SHMT2* [14], which are involved in nucleotide biosynthesis, likely diminishes the availability of nucleotides required for DNA replication and/or repair underling increases in DSBs and CIN, while reduced *GARS* [15] or *PIGS* [46] expression may indirectly induce CIN from defective translation or localization of proteins that normally function to maintain chromosome stability, respectively.

Of particular interest, *DSN1*, *NUF2* and *SKP1* silencing induced extensive chromosomal damage and shattering that is reminiscent of chromothripsis [47,48]. Chromothripsis is an emerging form of genome instability that results from a single catastrophic event inducing extensive DSBs that randomly reassemble in an oncogenic manner, for instance by creating oncogenic fusion genes or interrupting tumor suppressor genes [49]. Clinical studies have uncovered chromothripsis signatures in ~5% of all cancers, but they are most prevalent in soft tissue tumors including, liposarcomas (53.5%), fibrosarcomas (23.7%) and other sarcomas (22.9%) [50]. Although the underlying mechanisms accounting for chromothripsis remain largely unknown, emerging data suggest defects in DNA replication and repair may be critical etiological events. A number of mechanistic models exist to explain chromothripsis (reviewed in [28,35]). However, in the context of our findings, the “micronucleus model” is the most relevant. This model builds upon the observation that micronuclei form from chromosome mis-segregation events that typically arise from DSB repair defects. It further proposes that due to the limited amounts of DNA replication/repair machinery contained within a micronucleus, DNA replication occurs more slowly than in the primary nucleus and may be sensed as damaged DNA. Thus, a partially replicated chromosome, combined with inefficient DSB repair and the lack of a cell cycle arrest may enable the fragmented chromosome to ultimately fuse in a single event into a functional, yet highly rearranged chromosome. Our findings support this possibility and provide a potential mechanism by which this occurs. As indicated above, *DSN1*, *NUF2* and *SKP1* silencing induce increases in MN formation and extensive DSBs in multiple cell contexts. Furthermore, *SKP1* silencing corresponded with increases in replication stress and DSBs that are coincident with the mis-regulation of CCNE1 protein turnover. As CCNE1 is a prototypic cell cycle regulated protein required for the G1 to S-phase transition, DNA replication and genome stability (reviewed in [37]), our *SKP1* silencing data satisfy many of the mechanisms proposed within the micronucleus model. Thus, our data indicate that reduced *SKP1* expression induces chromothriptic events, a small subset of which may promote neoplastic transformation.

Reduced *SKP1* expression adversely impacts SCF complex formation and function and is expected to induce CIN through the resultant aberrant proteolytic degradation of target proteins. The finding that co-silencing *CCNE1* rescues ~50% of the aberrant phenotypes induced following *SKP1* silencing identifies CCNE1 as the key misregulated target protein; however, as co-silencing did not completely prevent the CIN phenotypes, additional SCF targets are likely involved that remain to be identified. Under normal conditions, CCNE1 promotes cell cycle progression by activating CDK2 (Cyclin Dependent Kinase 2) at the G1/S-phase boundary, but it also functions in histone biosynthesis, centriole duplication, apoptotic inhibition and DNA replication [51,52]. Genomic amplification of *CCNE1* occurs in many cancer types [41,42,43,44] and is an established driver of CIN, cellular transformation and cancer progression [39,45]. Further, constitutive *CCNE1* overexpression in fallopian tube secretory epithelial cells induced DNA damage, replication stress and CIN that corresponded with cellular transformation [45], while doxycycline induced overexpression in mice induced DNA damage, CIN and hepatocellular carcinoma [53]. Although previous studies have focused exclusively on the impact aberrant *CCNE1* expression has at the transcriptional level, our data show that proper temporal regulation at the protein level is also essential for genome stability. In fact, many of the aberrant phenotypes we observed following *SKP1* silencing and attributed to a lack of CCNE1 protein turnover, phenocopy those identified following genomic amplification or induced overexpression of *CCNE1*. Thus, we have established a novel, yet complementary mechanism whereby the lack of proteolytic degradation of CCNE1 stemming from a defective SCF complex induces replication stress, DSBs and CIN, which are enabling features of cancer. Our data further suggest that hypomorphic mutations in the remaining SCF complex member genes (i.e., RBX1, CUL1 and FBOX proteins) may also be pathogenic events, as similar outcomes on CCNE1 levels and CIN are expected to occur as a result of aberrant SCF formation and function. Finally, our findings may help explain the high prevalence of CIN in a subset of cancers lacking *CCNE1* amplification, but in which *SKP1*, or other SCF complex members are heterozygously or homozygously lost. In these cases, the accumulation of Cyclin E1 stemming from a lack of protein turnover due to reduced *SKP1* expression phenocopies genomic amplification of *CCNE1* by inducing CIN.

Overall, the results of this study have revealed a high level of conservation between yeast and human CIN genes and have identified critically important genes and biological processes that are central to preserving genome stability in humans. The reduced expression of 10 genes frequently observed in cancer each induce CIN and therefore may be significant, yet currently unappreciated drivers of neoplastic transformation and cancer progression. To explore this possibility, future studies should explore the clinical utility of all 148 CIN genes as novel diagnostic and/or prognostic biomarkers in various cancer types. Finally, CIN genes and the consequences of their aberrant expression in cancer may reveal genetic susceptibilities that can be therapeutically exploited within the clinic. For example, therapeutic strategies designed to exacerbate or target CIN (reviewed in [4]), such as synthetic lethal approaches can be used to leverage defects in CIN genes for highly specific killing of cancer cells [23]. Thus, the results gleaned from this study have shed insight into novel molecular determinants of CIN in cancer and have provided a wealth of CIN genes to explore in future fundamental, translational and health outcomes studies.

## 4. Materials and Methods

### 4.1. Cell Lines and Culture

The karyotypically stable human fibrosarcoma (HT1080) and immortalized (telomerase) normal skin fibroblast (hTERT) cell lines, were generously provided by Drs. J. Chubb (University College, London, UK) and C. P. Case (University of Bristol, Bristol, UK), respectively. HT1080 and hTERT cells were cultured in Dulbecco’s modified Eagle’s medium/High glucose media (HyClone, Logan, UT, USA) supplemented with 10% fetal bovine serum (FBS). The human epithelioid colorectal carcinoma (HCT116) cell line was purchased from American Type Culture Collection (Rockville, MD, USA), and cultured in modified McCoy’s 5A (HyClone) with 10% FBS. Cell lines were authenticated on the basis of recovery, viability, growth, morphology and spectral karyotyping as detailed elsewhere [54]. All cells were grown in a 37 °C humidified incubator with 5% CO_2_.

### 4.2. Identification of Candidate Human CIN Genes

From the list of 692 *Saccharomyces cerevisiae* CIN genes, Stirling et al. [5] identified 485 sequence-based and/or functional human orthologs. Of these, a total of 164 genes exhibiting the strongest meta-scores (i.e., repeatedly demonstrated a strong CIN phenotype) [5], but were not already established human CIN genes were selected as candidate CIN genes for screening.

### 4.3. Gene Silencing

A siGENOME library (Dharmacon, Lafayette, CO, USA) containing a pool of 4 distinct siRNA duplexes (6.25 pmol) in each well that target a specific candidate gene or in separate wells, positive and negative controls [6,7], was employed in the scQuantIM screen. Standard reverse transfection with DharmaFECT 2 transfection reagent (Dharmacon, Lafayette, CO, USA) was performed according to manufacturer’s instructions. Cells were seeded, allowed to grow for 4 (HCT116) to 6 days (hTERT) at 37 °C, fixed (4% paraformaldehyde) and counter-stained (Hoechst 33342) to visualize nuclei [7]. Subsequent direct tests employed ON-TARGETplus siRNA duplexes (Dharmacon, Lafayette, CO, USA) in a pooled format as detailed elsewhere [6]. Gene silencing was confirmed by Western blot 4 days post-transfection as described [23]. Membranes were blotted with the primary antibody at the indicated dilutions (Table 1) and visualized using secondary antibodies conjugated to horseradish peroxidase. Blots were imaged on a MyECL Imager (Thermo Scientific, Mississauga, ON, Canada) using standard chemiluminescence.

### 4.4. ScQuantIM

Images were acquired using a Cytation 3 Cell Imaging Multi-Mode Reader (BioTek, Winooski, VT, USA) equipped with a 16 bit, gray scale, charge-coupled device (CCD) camera and a 20× (0.45 numerical aperture) lens. A total of 9 non-overlapping (3 × 3 matrix) images were acquired from each well using the DAPI filter to visualize Hoechst 33342 (nuclei) and imported into Imaris v7.7.2 (Bitplane) image visualization software for analysis. Surface renderings of interphase nuclei (based on Hoechst staining) were generated in Imaris from which corresponding nuclear areas were determined. An XY boundary exclusion filter (<7 μm) was employed to remove partial nuclei along the image periphery, while inclusion filters were employed for area (250–2800 µm^2^) and mean Hoechst intensity (4500–5 × 10^4^ au) to eliminate small nuclear debris (i.e., apoptotic bodies) and mitotic cells, respectively. MN were assessed as detailed previously [55,56,57]. Briefly, MN were operationally defined as small (<1/3 the size of the nucleus), extra-nuclear Hoechst-stained bodies exhibiting no visible attachments with the primary nucleus. Accordingly, area (1.25–70 µm^2^) and Hoechst intensity center (>1 × 10^4^ au) filters were employed to automatically detect and enumerate micronuclei. NA and MN data were exported into Prism v6 (GraphPad) where two-sample Kolmogorov–Smirnov (KS) tests were performed on NA data and genes exhibiting a statistically significant change (*p*-value < 0.01) in cumulative NA distributions compared to siControl were identified as putative human CIN genes. Additionally, genes causing a significant increase in MN formation (greater than mean + 2 SD of siControl) were identified as putative CIN genes. Conditions with <50 interphase nuclei (within the 9 images) were excluded from analyses as they are predicted to induce death (i.e., essential gene) and/or cell cycle arrest. Representative images were exported into Photoshop CS6 (Adobe) where figure panels were assembled. Putative CIN genes were prioritized for subsequent validation based on the number of assays in which the gene was identified (i.e., ≥3 assays) and those that induced the strongest CIN phenotypes.

### 4.5. Gene Alterations and Outcome Analyses in Cancer

Publicly available genomic and mRNA expression data were freely obtained from TCGA network (https://portal.gdc.cancer.gov/) [19]. Genomic data from 12 common cancer types (breast, cervical, colorectal, glioblastoma, head and neck, renal, liver, lung, ovarian, pancreatic, prostate and uterine) were extracted, analyzed and visualized using cBioPortal (www.cbioportal.org) and onco-query commands (HETLOSS, HOMDEL, AMP and GAIN) [18]. Raw mRNA expression data and survival outcomes were exported from TCGA and visualized in The Human Protein Atlas ((https://www.proteinatlas.org/) [20]. The threshold between high and low mRNA expression was determined as described elsewhere [20]. Briefly, for each gene, the threshold was selected as the mRNA expression level between the 20th and 80th percentile that results in the lowest log-rank *p*-value in the survival analysis comparing patients with high or low mRNA expression. Based on this analysis, Kaplan–Meier curves were generated and statistical analyses (log-rank tests) were performed (*p*-value < 0.05 is significant). All figures were assembled in Photoshop CS6 (Adobe).

### 4.6. Mitotic Chromosome Spreads and Chromosome Enumeration

Subconfluent hTERT and HCT116 cells were mitotically enriched using KaryoMAX colcemid (0.1 mg/mL; Gibco), treated with hypotonic solution (75 mM KCl), fixed (3:1 Methanol:Acetic acid), and mounted on slides (DAPI mounting media) as described [6]. A minimum of 100 spreads/condition were imaged and manually enumerated using ImageJ software. Small-scale numerical changes (i.e., involving <15 chromosomes), large-scale numerical changes (i.e., involving ≥15 chromosomes), structural (chromosome breakages or decompaction) and cohesion defects, which are indicative of numerical and/or structural CIN, were visually assessed. Statistical differences in the cumulative distribution frequencies of chromosome complements were assessed relative to control using two-sample KS tests (Prism).

### 4.7. Indirect Immunofluorescent Labeling and Analysis

Cells were fixed (4% paraformaldehyde), permeabilized (0.5% Triton-X100 in PBS), immunofluorescently labeled, counterstained with DAPI, and images were collected as detailed elsewhere [54]. Table 1 provides a list of the antibodies and dilutions employed. To assess replication stress, cells were treated with a positive control (hydroxyurea; 10mM, 3 h) and immunofluorescently labeled for Replication Protein A (RPA). To assess increases in DNA damage, cells were treated with a positive control (bleomycin; 0.1 μg/mL, 2 h) and immunofluorescently labelled for γH2AX, a surrogate marker of DSBs. ScQuantIM was employed as described previously [54], with constant exposure times employed throughout the image acquisition process. The number of γH2AX foci/nuclei was manually assessed in Imaris, and the standard definition of ≥5 γH2AX foci/nuclei employed as the minimum threshold required to identify a positive nucleus.

## 5. Conclusions

Cancer remains a devastating disease throughout the world, highlighting an urgent need for innovative strategies to better combat the disease. To develop highly selective therapeutic strategies requires new insight into the abnormal genes and pathways driving disease development and progression. Chromosome instability (CIN) or constantly evolving changes in chromosome numbers is a form of genome instability associated with disease development, evolution, spread and drug resistance, yet the mutated genes giving rise to CIN remain largely unknown. Here, we employ a microscopy based approach and identify 148 genes that when silenced induce CIN. Using a series of complementary genetic, biochemical and microscopy-based approaches we focus on 10 genes and confirm each as a novel CIN gene. More focused work on one gene (*SKP1*) also identified the mechanisms by which it induces CIN. Collectively, this study has identified novel CIN genes that when mutated may be significant contributors to disease development and progression. As such, future work can now focus on developing therapeutic strategies that selectively exploit defects in these genes occurring in many different cancer types.

## Figures and Tables

**Table 1:** List of antibodies employed in this study.

Target Protein ^1^	Species	Catalog No.	[WB] ^2^	[IIF] ^3^
α-Tubulin *	Mouse	Abcam, ab7291	1:4000	-
ARL2	Rabbit	Abcam; ab183510	1:5000	-
BUB3	Rabbit	Abcam; ab4180	1:5000	-
CCNE1	Rabbit	Abcam; ab133266	1:5000	1:200
Cyclophilin B *	Rabbit	Abcam; ab16045	1:50,000	-
DSN1	Rabbit	Thermo Fisher; PA534879	1:1000	-
GARS	Rabbit	Abcam; ab42905	1:5000	-
GART	Rabbit	Abcam; ab169550	1:5000	-
γH2AX	Rabbit	Abcam; ab2893	-	1:1000
NUF2	Rabbit	Abcam; ab176556	1:20,000	-
PIGS	Rabbit	Abcam; ab113817	1:5000	-
RPA32/RPA2	Mouse	Abcam; ab2175	-	1:200
SHMT2	Rabbit	Abcam; ab180786	1:1000	-
SKP1	Mouse	Abcam; ab124473	1:5000	-
SPC24	Rabbit	Abcam; ab157184	1:5000	-
Anti-Rabbit HRP	Goat	Jackson Immunoresearch; 111-035-114	1:10,000	-
Anti-Mouse HRP	Goat	Jackson Immunoresearch; 115-035-146	1:10,000	-
Anti-Rabbit AlexFluor488	Goat	Abcam; ab150081	-	1:200
Anti-Mouse Cy3	Goat	Abcam; ab150117	-	1:200

^1^ Proteins listed with an asterisk represent Western blot loading controls. HRP = horse radish peroxidase. ^2^ Concentration of antibody employed for Western blot analyses. ^3^ Concentration of antibody employed for indirect immunofluorescence. * identifies protein loading controls.

## Supplementary Materials

The following are available online at https://www.mdpi.com/2072-6694/12/3/531/s1. Figure S1: Reduced Expression of all 10 Putative CIN Genes Corresponds with Poor Overall Patient Survival in Various Cancer Types, Figure S2: Western Blots Showing Efficient Gene Silencing, Figure S3: Gene Silencing Induces Chromosomal Defects in HCT116 Cells, Figure S4: Raw data of western blots presented in Figure 5, Table S1: Cross-species Approaches Yield 164 Human Candidate CIN Genes, Table S2: KS Tests Reveal Significant Changes in NA Following Silencing in hTERT, Table S3: Micronucleus Enumeration Identifies Putative CIN Genes in hTERT, Table S4: KS Tests Reveal Significant Changes in NA Following Silencing in HT1080, Table S5: Micronucleus Enumeration Identifies Putative CIN Genes in HT1080, and Table S6: CIN Phenotype Validation for Putative Human CIN Genes.
